# Impact of Specific Pattern Mutations on Plaque Formation and Attenuation Properties of Dengue Virus Serotype 3

**DOI:** 10.1155/av/9239020

**Published:** 2026-09-29

**Authors:** Panumas Sun-Arlee, Surat Punyahathaikul, Ekkarat Rodpai, Kunjimas Ketsuwan, Duangnapa Wanlayaporn, Sasiporn Ruangdachsuwan, Jukrapun Komaikul, Thitigun Jaimipuk, Sanjira Juntarapornchai, Chompunuch Boonarkart, Anan Jongkaewwattana, Sutee Yoksan, Prasert Auewarakul, Promsin Masrinoul

**Affiliations:** ^1^ Graduate Program in Molecular Medicine, Faculty of Science, Mahidol University, Bangkok, Thailand, mahidol.ac.th; ^2^ Center for Vaccine Development, Institute of Molecular Biosciences, Mahidol University, Nakhon Pathom, Thailand, mahidol.ac.th; ^3^ Department of Microbiology, Faculty of Medicine Siriraj Hospital, Mahidol University, Bangkok, Thailand, mahidol.ac.th; ^4^ Virology and Cell Technology Laboratory, National Center for Genetic Engineering and Biotechnology (BIOTEC), National Science and Technology Development Agency, Pathumthani, Thailand, nstda.or.th

**Keywords:** attenuation phenotype, dengue virus serotype 3, live attenuated vaccine, mouse neurovirulence

## Abstract

Dengue fever is a major public health problem in several countries, including Thailand. The development of an effective dengue vaccine against all four serotypes is important for controlling the disease. An attenuated dengue‐3 vaccine candidate, namely, DENV3‐MBU‐1, was derived from full‐length cDNA‐assembled segments based on the sequence of a prototype DENV3 vaccine candidate originating from the DENV3 strain 16562, which was passaged 30 times in primary green monkey kidney cells and four times in primary dog kidney cells. Compared to the parental DENV3‐16562 sequence, this DENV3‐MBU‐1 contains seven nonsynonymous mutations in the premembrane (prM), envelope (E), and nonstructural proteins (NS2A, NS3, NS4). In this study, we aimed to characterize the mutations contributing to attenuation by generating different DENV3 mutants and evaluating their properties, including plaque formation and neurovirulence in mice. The DENV3‐MBU‐1 mutants, in which the NS proteins were reverted to those of the parental virus, exhibited an altered phenotype, manifested by changes in plaque size and virulence, resembling that of the parental virus. This suggests the important role of the NS segment in attenuation. Interestingly, the mutants of DENV3‐MBU‐1, in which only the structural proteins, but not the NS proteins were altered, showed changes in phenotype toward increased plaque size and neurovirulence. This suggests that attenuation may be influenced by specific combinations of mutations rather than single‐point mutations. These findings, along with the attenuated DENV3 viruses generated, provide useful information for the development of a dengue vaccine.

## 1. Introduction

Dengue virus (DENV) is one of the most serious human pathogens and belongs to the genus Orthoflavivirus and the family Flaviviridae, along with other important human pathogens such as Zika virus and Japanese encephalitis virus (JEV) [1, 2]. DENV consists of four serotypes: dengue virus serotype 1 (DENV1), DENV2, DENV3, and DENV4 [3]. However, all four serotypes share the same genomic structure and organization: A linear, single‐stranded 11‐kb RNA flanked by 5′ and 3′ untranslated regions (UTR) and encoding 3,386 amino acids translated as a large open reading frame—capsid (C), premembrane (prM), envelope (E), nonstructural protein 1 (NS1), NS2A, NS2B, NS3, NS4A, NS4B, and NS5. The three structural and seven NS proteins are processed from a precursor polyprotein by host and viral proteases and enzymes [4]. The four serotypes have an amino acid identity of about 60%–75% [5].

Infection with any of the four serotypes remains a serious public health problem and results in a wide clinical spectrum of disease ranging from undifferentiated fever and dengue fever to life‐threatening dengue hemorrhagic fever (DHF) and hypovolemic shock or dengue shock syndrome (DSS) [6]. Although approximately 75% of dengue‐infected individuals are asymptomatic, fewer than 5% of infected patients develop severe disease [7]. Due to the different levels of sequence similarities between serotypes, immune responses to genetically and antigenically closely related viruses are complex. The host immune response to a primary infection with one serotype can elicit both specific monotypic and partially cross‐reactive heterotypic responses. These suboptimal, cross‐reactive antibodies can increase the risk of antibody‐dependent enhancement (ADE) by binding to the virus and facilitating the entry of the virus–antibody complex via the Fcγ receptor, thereby increasing viral replication. This makes vaccine development particularly challenging, as balanced protective immunity against all four serotypes must be induced [8, 9].

Several types of DENV vaccines have been developed, including live attenuated vaccines (LAVs), subunit vaccines, and inactivated vaccines [10]. Two LAV‐DENV vaccines are currently licensed in numerous countries. CYD‐TDV (Dengvaxia^®^, Sanofi Pasteur), a live tetravalent chimeric DENV vaccine using a yellow fever virus backbone, is approved for a three‐dose vaccination regimen for individuals aged 9 to 45 years. However, Dengvaxia was found to partially protect children who had previous DENV infections, but it increased the risk of severe breakthrough DENV disease in children who were seronegative [11]. Moreover, vaccine efficacy varies by serotype: 54.7% for DENV1, 43.0% for DENV2, 71.6% for DENV3, and 76.9% for DENV4 [12, 13]. Recently, TAK‐003 (Qdenga^®^, Takeda), a live recombinant chimeric vaccine with structural prM and E proteins of dengue on a DENV2 primary dog kidney 53 (PDK53) backbone, has been approved for a two‐dose vaccination schedule. The overall vaccine efficacy for the primary endpoint is 80.2% at a 12‐month follow‐up. However, after 4 to 5 years of follow‐up, efficacy was demonstrated against all four DENV serotypes in dengue‐seropositive participants and against DENV1 and DENV2 in dengue‐seronegative participants. Data suggest a lack of efficacy against DENV3, and the low incidence of DENV4 precluded an evaluation of efficacy in dengue‐seronegative participants [14]. Due to this limited evidence, the World Health Organization’s (WHO) Strategic Advisory Group of Experts (SAGE) recommended introducing the vaccine in regions with a high burden of dengue disease and high transmission rates to maximize public health benefits and minimize potential risks in seronegative populations [15]. These results underline the need for continued development to improve the efficacy of the dengue vaccine.

A LAV‐DENV candidate was developed at the Center for Vaccine Development, Institute of Molecular Biosciences, Mahidol University in Thailand. In contrast to chimeric vaccines, this prototype was produced by multiple passages of DENV in nonhuman animal cells, thereby reducing its virulence. This approach was applied to all four serotypes. Selected passaged viruses were tested for immunogenicity and attenuation. For example, the DENV3 vaccine strain 16562 was passaged 30 times in primary green monkey kidney (PGMK) cells, followed by four additional passages in PDK cells, resulting in the PGMK30/PDK4 strain, which exhibits potentially attenuated characteristics including small plaque size and low neurovirulence in suckling mice. This vaccine has been further developed as a vaccine seed and is part of the live tetravalent dengue vaccine, KD‐382 (KM Biologics). Studies in nonhuman primates have shown that this vaccine elicits potent neutralizing antibodies and provides protective capabilities, with no detectable viral RNA in serum from either seronegative or seropositive dengue monkeys [16, 17]. Additionally, Phase I clinical trials have shown that the KD‐382 vaccine is safe and well tolerated in healthy adults. A 100% seroconversion rate was achieved at an early stage and a neutralizing antibody response was induced against all four serotypes, with the antibody response persisting in most subjects for a period of 1 year after administration of a single dose [18].

Since the DENV3 vaccine prototype exhibits attenuation characteristics, the molecular markers responsible for this attenuation have not yet been identified. To address this, we created a recombinant version, DENV3‐MBU‐1, through RT‐PCR amplification and assembly of segments from the vaccine prototype DENV3‐PGMK30/PDK4. Although DENV3‐MBU‐1 has not been tested in clinical trials, it has shown small plaque formation and low neurovirulence in suckling mice. Using reverse genetics, we aim to characterize the attenuating mutations in DENV3‐MBU‐1. Analysis of the complete genome sequence identified seven nonsynonymous mutations: four in structural proteins and three in NS proteins. Using a reverse genetics approach, the mutations in the vaccine candidate strain were reverted to the parental strain. The mutants were then screened for attenuation by plaque formation and neurovirulence testing in mice, enhancing our understanding of attenuating mutations for further vaccine development.

## 2. Materials and Methods

### 2.1. Cells and Viruses

African green monkey kidney cells, or Vero cells (ATCC^®^ CCL‐81™), were cultured at 37°C with 5% CO_2_ in Minimum Essential Medium (MEM) (Gibco, Thermo Fisher Scientific, MA, USA), supplemented with 10% heat‐inactivated fetal bovine serum (FBS) (Gibco, Thermo Fisher Scientific, MA, USA) and 1% Antibiotic/Antimycotic (Anti/Anti) (Gibco, Thermo Fisher Scientific, MA, USA). Human embryonic kidney cells (HEK‐293T; ATCC^®^ CRL‐11268™) and Rhesus monkey kidney cells (LLC‐MK2; ATCC^®^ CCL‐7™) were grown in Dulbecco’s modified Eagle’s medium (DMEM) (Gibco, Thermo Fisher Scientific, MA, USA), 10% heat‐inactivated FBS, and 1% Anti/Anti at 37°C with 5% CO_2_. The human monocytic leukemia THP‐1 cell line was grown in RPMI 1640 (Gibco, Thermo Fisher Scientific, MA, USA) with 10% heat‐inactivated FBS and 1% penicillin/streptomycin (100 units/mL) at 37°C with 5% CO_2_. *Aedes albopictus* larvae cell (C6/36) was cultured at 28°C–32°C without CO_2_ in Leibovitz L‐15 media (Sigma‐Aldrich Co., St. Louis, MO, USA) supplemented with 10% heat‐inactivated FBS (Gibco) and 1% penicillin/streptomycin (100 units/mL).

The parental dengue virus serotype 3 (DENV3‐16562) and DENV3 strain DENV3/PGMK30/PDK4 (DENV3‐MBU‐1) were propagated in Vero cells in culture medium without FBS for 60 min before adding MEM with 2% FBS and incubated at 37°C in 5% CO_2_ until cytopathic effects (CPEs) were observed. The culture medium containing viruses was harvested from the culture supernatant and stored at −80°C for further experiments. Virus titer was determined by plaque assay.

### 2.2. Construction of DENV3 Template Plasmid and the Subgenomic Fragments

The template plasmid containing the full‐length DENV3 genome was constructed by assembling subgenomic fragments. Briefly, viral RNA was extracted from culture supernatants of DENV3‐16562 using the High Pure RNA Isolation Kit (Roche Molecular Systems, Inc., USA) according to the manufacturer’s instructions. cDNA synthesis was performed using the ImProm‐II™ reverse transcriptase kit (Promega Corporation, Madison, WI, USA). The synthesized cDNA was used as template for amplifying the genome fragments using the Platinum™ Taq DNA Polymerase High Fidelity kit (Thermo Fisher Scientific, USA) according to the manufacturer’s instructions with primers spanning five fragments with overlapping regions of 15–30 nucleotides: Fragment 1 (FR1; nucleotide position 1 to 2121), FR2 (nucleotide position 2090 to 4796), FR3 (nucleotide position 4766 to 6587), FR4 (nucleotide position 6557 to 8522), and FR5 (nucleotide position 8498 to 10,696). The list of primers used for PCR amplification is shown in Supporting Table S1. Each fragment was purified using GenepHlow™ Gel/PCR Kit (Geneaid Biotech Ltd., USA) according to the manufacturer’s instructions. Each fragment was then assembled and cloned into a modified pSMART‐BAC vector containing a cytomegalovirus (CMV) promoter and a BGH terminator sequence using In‐Fusion® HD Cloning Kit (Takara Bio Inc., Shiga, Japan) according to the manufacturer’s instructions. The assembled product was ligated and transformed into NEB10β competent cells. The plasmid containing assembled genomic DNA was sent for sequencing. However, obtaining the complete genome of DENV3 proved to be difficult, probably due to the instability of the DENV3 genome in bacteria. Nevertheless, this plasmid, referred to as pDENV3‐ΔFL, served as a template for the generation of other mutant forms of DENV3. Mutations were introduced using mutagenic primers to correct sequences and construct additional DENV3 mutants, all based on the pDENV3‐ΔFL template. The list of primers used for mutant construction is shown in Supporting Table S2. PCR products were purified according to the previously mentioned protocol.

### 2.3. Sequence Analysis

The nucleotide sequences of DENV3 were analyzed using bioinformatics tools. The chromatograms were analyzed with FinchTV 1.4.0 [19]. The nucleotide and amino acid sequences were aligned with the parental strain and other mutant strains using the multiple sequence alignment program BioEdit Sequence Alignment Editor [20].

### 2.4. Generation of DENV3 and Designed Mutant Viruses by Subgenomic Fragment Assembly

The purified PCR fragments containing the designed mutations were assembled using the In‐Fusion® HD Cloning Kit (Takara Bio Inc., Shiga, Japan), followed by ligation using the Quick Ligation™ Kit (New England BioLabs Inc., MA, USA). The assembled PCR fragments were transfected into cocultured Vero:HEK‐293T cells at 60%–70% confluence at a ratio of 1:3 or Vero cells in 6‐well plates using Lipofectamine® 3000 reagent (Invitrogen, Thermo Fisher Scientific, MA, USA). The cells were incubated at 37°C in 5% CO_2_ for 8 h, after which the supernatants were replaced with 2% FBS Opti‐MEM® I Reduced Serum Medium and continued to be incubated at 37°C in 5% CO_2_ for 7 to 14 days. On Days 7 and 14 after transfection, the culture fluids were passaged into Vero cells. The culture supernatants were harvested by centrifugation. The viruses were sent for sequencing to determine their identity.

### 2.5. Plaque Assay

Plaque assays were performed using LLC‐MK2 cells in 6‐well plates. Cells were infected with 10‐fold serially diluted virus at 37°C in 5% CO_2_ for 60 min. Infected cells were overlaid with 1.5 mL of overlay MEM medium containing 1.2% carboxymethyl cellulose, 1% FBS, 1X antibiotics, and neutral red and incubated for 7 days at 37°C in 5% CO_2_. The plaques were counted, and the plaque size was determined.

Plaque size was measured by randomly selecting thirty plaques using Microsoft software. The diameter of each plaque was then magnified, measured, and calculated in relation to the diameter of the well to determine the actual plaque size. Plaque diameters less than 1.0 mm were categorized as small plaque size (S), and diameters greater than 2.0 mm were categorized as large plaque size (L), while sizes between 1.0 mm and 2.0 mm were categorized as medium plaque size (M). Differences in mean plaque diameters of mutant viruses compared to DENV3‐16562 and DENV3‐MBU‐1 viruses were statistically determined by Student′s t‐tests, with significance achieved at a *p*‐value ≤ 0.05.

### 2.6. Mouse Neurovirulence

All animal experiments were conducted with the approval of the Institute of Molecular Biosciences Animal Care and Use Committee, Mahidol University (IMB‐ACUC 2017/007). The experiments were carried out in accordance with ethical principles and guidelines for the use of animals by the National Research Council of Thailand and the Animal Research: Reporting of In Vivo Experiments (ARRIVE) guidelines. Briefly, the pregnant ICR mice were obtained from Nomura Siam International (Thailand). Two‐day‐old ICR mice were injected intracranially (i.c.) with normal saline solution (NSS) for the control group and with 1000 pfu/mouse of either DENV3 or virus mutants. The injected mice were observed for 21 days to determine mortality. Euthanasia was performed using isoflurane overdose. Animals that died within 3 days postinjection due to inoculation were excluded from the analysis. The number of surviving mice was used to calculate the average survival time (AST) using SPSS Version 16.0. Survival rates and days postinjection were plotted, and differences in survival rates between groups were analyzed by Log‐rank (Mantel–Cox) tests in GraphPad Prism Version 8.0.1. Statistical significance was defined as a *p*‐value less than 0.05.

### 2.7. Immunofluorescence Assay

LLC‐MK2 cells were infected with DENV3 viruses at a multiplicity of infection (MOI) of 0.2 for 2 days. Cells were fixed with 4% formaldehyde in PBS for 20 min at room temperature, followed by three washes with PBS. The cells were then blocked with 1% bovine serum albumin (BSA) in PBS for 1 h at room temperature and washed once with PBS. Permeabilization was performed using 0.1% Triton X‐100 in PBS for 10 min, followed by three PBS washes. Cells were incubated with anti–double‐stranded RNA (anti‐dsRNA) (Jena Bioscience) for 1 h. After three washes with PBS, cells were incubated with Alexa Fluor–conjugated goat anti‐mouse IgG secondary antibody for 1 h in the dark. Following three additional PBS washes, nuclei were counterstained with 4′,6‐diamidino‐2‐phenylindole (DAPI) (ServiceBio; final concentration, 1 μg/mL) for 15 min. Fluorescence images were subsequently acquired using the Operetta confocal laser scanning (CLS) microscope (PerkinElmer).

### 2.8. Kinetics of DENV3 Mutants in THP‐1

THP‐1 cells were infected with DENV3 at a MOI of 2 in triplicate. Culture fluids were harvested daily for five consecutive days postinfection and stored at −80°C until use. Viral titers were determined by plaque assay.

### 2.9. RNA Extraction and Real‐Time RT‐PCR

DENV3‐infected THP‐1 cells were collected on Day 2 postinfection to analyze viral genomic RNA and interferon (IFN) response gene expression using RT‐qPCR. Briefly, total cellular RNA was extracted using TRIzol reagent (Invitrogen, USA). To assess the viral genomic RNA, the assay utilized primers and a TaqMan probe targeting the highly conserved 3′UTR of the DENV genome, as previously described [21]. The primer sequences were as follows: forward primer (3UTR_DENV_F) 5′‐GGTTAGAGGAGACCCCTCCC‐3′, reverse primer (3UTR_DENV_R1‐3) 5′‐GGCGYTCTGTGCCTGGA‐3′, and the probe (3UTR_DENV_P) 5′‐FAM‐CAGGATCTCTGGTCTYTCCCAGCGT‐BHQ1‐3′. For the quantification of viral RNA, a plasmid containing the DENV3 3′UTR was used as the standard, and the results were interpreted as viral RNA copies/mL.

To determine the IFN response gene expression, one‐step RT‐qPCR was performed using the qPCRBIO SyGreen® 1‐Step Kit (PCR Biosystems, UK) in the LightCycler® 480 (Roche) following the kit’s instructions. The following primers were used to detect the expression of specific genes: IFN‐α forward and reverse [22]; 2‐5‐oligoadenylate synthetase 1 (OAS1) forward and reverse [23]; E14134 and E14135 for glyceraldehyde‐3‐phosphate dehydrogenase (GAPDH) mRNA [24]. GAPDH was used as an internal control, and relative gene expression was compared between wild‐type virus‐infected and mutant virus‐infected samples using the 2^−ΔΔCT^ method [25]. Differences in the mean relative expression of mutant viruses compared to DENV3‐16562 were statistically analyzed by Student′s t‐test in SPSS Version 16.0, with significance defined at *p*‐value ≤ 0.05.

## 3. Results

### 3.1. Comparison of the Genomic Sequences Between DENV3‐MBU‐1 and the Parental DENV3 Strain Revealed Seven Nonsynonymous Mutations

A DENV3‐MBU‐1 was generated from RT‐PCR–amplified genome segments of the dengue 3 vaccine prototype DENV3‐16562/PGMK30/PDK4, which was assembled into a full‐length genome by Gibson assembly. The virus recovered from the infectious clone showed signs of attenuation as evidenced by the production of smaller plaques compared to the parental DENV3‐16562 strain. This characteristic of the virus was notably shown to elicit a good antibody response in monkeys. Subsequently, the complete genome sequence of DENV3‐MBU‐1 was compared with the parental DENV3‐16562 virus to identify the potential determinants contributing to the attenuation of viral virulence. Of the differences observed, twelve nucleotide substitutions were observed between DENV3‐MBU‐1 and the parental DENV3‐16562. Of these, five were synonymous G1813A, C2140T, G3481A, G6069A, and A6688G, and the remaining seven were nonsynonymous, A719T, A1838G, G1913A, G2106A, T3725C, C4781A, and G6572A, located in prM, E, NS2A, NS3, and NS4A, as shown in Table 1.

**TABLE 1:** Nucleotide and amino acid differences between DENV3‐MBU‐1 and DENV3‐16562.

No.	Genome position	aa position (protein position)	Gene region	DENV3‐MBU‐1	DENV3‐16562	Type of mutations
				Codon (aa)	Codon (aa)	
1	719	209 (prM‐95)	prM (M)	**T**TA (L)	**A**TA (I)	NS
2	1813	573 (E‐294)	E (EDII)	AA**A** (K)	AA**G** (K)	S
3	1838	582 (E‐302)	E (EDIII)	**G**GT (G)	**A**GT (S)	NS
4	1913	607 (E‐327)	E (EDIII)	**A**AA (K)	**G**AA (E)	NS
5	2106	671 (E‐392)	E (EDIII)	A**G**G (R)	A**A**G (K)	NS
6	2140	682 (E‐403)	E (EDIII)	GC**T** (A)	GC**C** (A)	S
7	3481	1129 (NS2A‐1)	NS2A	AA**A** (K)	AA**G** (K)	S
8	3725	1211 (NS2A‐86)	NS2A	**C**TC (L)	**T**TC (F)	NS
9	4781	1563 (NS3‐90)	NS3	**A**AA (K)	**C**AA (Q)	NS
10	6094	2000 (NS3‐527)	NS3	AA**A** (K)	AA**G** (K)	S
11	6572	2160 (NS4A‐68)	NS4A	**A**CA (T)	**G**CA (A)	NS
12	6688	2198 (NS4A‐105)	NS4A	GC**G** (A)	GC**A** (A)	S

*Note:* NS, nonsynonymous mutation; S, synonymous mutation.

Abbreviation: aa, amino acid.

### 3.2. Generation of DENV3 With Single and Multiple Identified Mutations

Several strategies were employed to generate DENV3 using reverse genetics. Our initial attempt was to construct a plasmid with the complete genome of DENV3 containing a CMV promoter and BGH terminator sequences. This was achieved by assembling overlapping fragments of DENV3 and transforming them into NEB10β bacterial cells. However, due to a nucleotide deletion at Position 2472 in the NS1 gene, we were unable to generate the full‐length DENV3 genome; therefore, we generated a plasmid with a nucleotide deletion called pDENV3‐ΔFL. Consequently, this plasmid cannot serve as a full‐length infectious cDNA clone. As an alternative strategy, we used this plasmid to generate DENV3 by infectious subgenomic amplicon assembly. This required us to design primers to correct the deletion and introduce mutations in specific genes to change the mutant amino acid of DENV3‐MBU‐1 back to wild type based on pDENV3‐ΔFL. The PCR fragments, each containing single or multiple point mutations, were amplified, assembled, and recovered in coculture of Vero:HEK‐293T. DENV3 production was determined by plaque assay. To investigate the effects of specific mutations identified in DENV3‐MBU‐1 on attenuation properties, a series of mutant viruses were produced. These included NS‐MBU‐1 and S‐MBU‐1, which contained NS and structural genes, respectively, similar to those of DENV3‐MBU‐1. Additionally, mutant viruses named MBU‐1‐1.1m to MBU‐1‐1.7m were developed, each containing one of seven mutations identified in MBU‐1. Other mutants contained combinations of several mutations. A total of 14 mutant viruses were successfully generated, as shown in Figure 1, Table 2, and Supporting Figures S1 and S2. However, some mutants, particularly MBU‐1‐1.3m and MBU‐1‐1.5m, could not be generated despite several attempts. The mutations in the successfully recovered viruses were confirmed to be correct by whole‐genome sequencing analysis, and no unintended mutations were detected.

**FIGURE 1:**
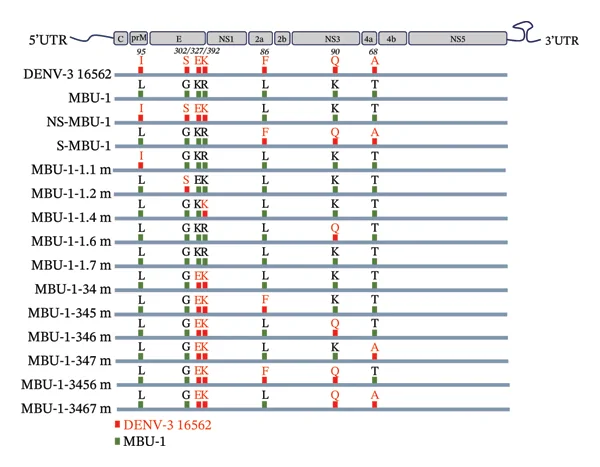
Schematic representation of the recombinant DENV3 viruses generated and evaluated in this study. The genomic organizations of each virus are shown, including the structural and nonstructural protein‐coding regions. Amino acid substitutions introduced into each recombinant construct are indicated at their corresponding positions. At variable amino acid positions, residues derived from DENV3‐MBU‐1 are shown in red, whereas those derived from DENV3‐16562 are shown in green.

**TABLE 2:** Mutant viruses and corresponding nucleotides at seven positions.

Nucleotide position	719	1838	1913	2106	3725	4781	6572
Gene	prM	E	E	E	NS2A	NS3	NS4A
DENV3‐16562	**A**	**A**	**G**	**A**	**T**	**C**	**G**
DENV3‐MBU‐1	T	G	A	G	C	A	A
NS‐MBU‐1	**A**	**A**	**G**	**A**	C	A	A
S‐MBU‐1	T	G	A	G	**T**	**C**	**G**
MBU‐1‐1.1 m	**A**	G	A	G	C	A	A
MBU‐1‐1.2 m	T	**A**	A	G	C	A	A
MBU‐1‐1.3 m	No virus produced						
MBU‐1‐1.4 m	T	G	A	**A**	C	A	A
MBU‐1‐1.5 m	No virus produced						
MBU‐1‐1.6 m	T	G	A	G	C	**C**	A
MBU‐1‐1.7 m	T	G	A	G	C	A	**G**
MBU‐1‐34 m	T	G	**G**	**A**	C	A	A
MBU‐1‐345 m	T	G	**G**	**A**	**T**	A	A
MBU‐1‐346 m	T	G	**G**	**A**	C	**C**	A
MBU‐1‐347 m	T	G	**G**	**A**	C	A	**G**
MBU‐1‐3456 m	T	G	**G**	**A**	**T**	**C**	A
MBU‐1‐3467 m	T	G	**G**	**A**	**T**	A	**G**

*Note:* bold = reverted to the parental nucleotide.

### 3.3. Characteristics of Mutant Viruses

A common characteristic of attenuation in viruses is the formation of smaller plaque sizes. To investigate the phenotypes of the generated mutant viruses, we evaluated their phenotypic characteristics primarily through plaque assays. We compared the plaque sizes of all mutant viruses with those of the parental DENV3‐16562 and the recombinant live attenuated DENV3‐MBU‐1. Our findings indicated that mutations, whether single or multiple, affected the plaque size.

To further investigate the role of structural and NS genes in attenuation, we analyzed the phenotypes of S‐MBU‐1 and NS‐MBU‐1 in comparison with the parental DENV3‐16562 and DENV3‐MBU‐1. The results showed that S‐MBU‐1 formed significantly larger plaques than the parental DENV3‐16562, with an average plaque size of 2.09 ± 0.42 mm, suggesting that the alteration of the NS protein in DENV3‐MBU‐1 influences plaque size formation. In addition, NS‐MBU‐1 showed smaller plaques (0.88 ± 0.19 mm) compared to the parental strain, with a size comparable to that of the attenuated DENV3‐MBU‐1 (Figure 2). Since NS‐MBU‐1 contains NS proteins similar to those of DENV3‐MBU‐1 and structural proteins similar to those of the parental virus and had similar plaque sizes to DENV3‐MBU‐1, this emphasizes the importance of the NS proteins of DENV3‐MBU‐1 in reducing plaque size, highlighting their essential role in attenuating the virus.

**FIGURE 2:**
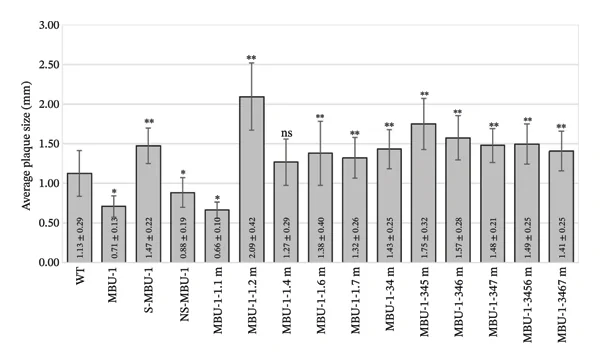
Average diameter (mm) of plaque produced. Bar graph representations of average diameters of plaques produced by viruses (parental and mutants). The standard deviation is indicated as error bars. The plaque assay was performed in a 6‐well plate. The average diameter was calculated from a random selection of thirty plaque spots per virus. The calculation was proportional to the average plaque well diameter. Error bars indicate standard deviation. *p*‐values were calculated using a t‐test, *p* < 0.05 relative to WT (parental). (^∗^) indicates significantly smaller, (^∗∗^) indicates significantly larger, and “ns” indicates nonsignificant differences.

We also investigated the effects of single mutations in the attenuated DENV3‐MBU‐1, which were reverted to the parental DENV3‐16562, based on plaque formation. The recombinant mutant viruses, MBU‐1‐1.1 m to MBU‐1‐1.7 m, were generated, and their plaque sizes were compared. Since the NS mutations appear to contribute to the attenuation phenotype, we first examined the effects of each mutation on the NS protein. The mutant viruses MBU‐1‐1.6 m and MBU‐1‐1.7 m, which have similar sequences to DENV3‐MBU‐1 except for the NS3 (K90Q) and NS4A (T68A) amino acid substitutions, respectively, exhibited average plaque sizes of 1.38 ± 0.40 mm and 1.32 ± 0.26 mm. These plaque sizes were not significantly different from those of the parental DENV3‐16562 virus (1.13 ± 0.29 mm). However, we were unable to rescue a virus with a single reversion in NS2A (MBU‐1‐1.5 m); the contribution of this mutation in NS2A could not be determined. Since the single mutations in NS3 or NS4A were unable to maintain a small plaque size, this suggests that a combination of mutations, rather than a single mutation in the NS protein, is required to reduce plaque size.

In addition, we investigated the effects of individual mutations in the structural proteins of the mutant viruses. The virus MBU‐1‐1.1 m, which contains the prM (L95I) mutation, had a small plaque size, averaging 0.66 ± 0.10 mm, similar to that of DENV3‐MBU‐1. This indicates that the prM mutation alone has no significant effect on plaque size formation. Interestingly, MBU‐1‐1.2 m, which has a similar sequence to DENV3‐MBU‐1 except for a mutation at Position E (G302S), significantly increased plaque size to 2.09 ± 0.42 mm, more than all other mutations observed. Although it contains three mutations in the NS protein like DENV3‐MBU‐1, the change at this position resulted in a large plaque phenotype, indicating a significant role in viral replication and plaque formation.

The effects of other mutations were less conclusive; MBU‐1‐1.3 m was not rescued, and MBU‐1‐1.4 m showed a trend toward a larger plaque (1.27 ± 0.29 mm), although not statistically significant (Figure 2). However, the double mutations at Positions K327E and E‐R392K in the envelope protein, designated MBU‐1‐34 m, were successfully produced. Simultaneous reversal of these points also resulted in significantly increased plaque size (1.43 ± 0.25 mm), suggesting that at least one of the mutations contributes to reduced plaque formation. As the individual mutation MBU‐1‐1.4 m did not significantly increase plaque size, this suggests that the mutation MBU‐1‐1.3 m or the combination of the two might play a greater role.

Furthermore, mutant viruses were created to combine different changes in structural and NS protein mutations. All led to an increase in plaque size, more so than when the NS mutations were reverted individually. This also shows the important role of the structural proteins in virus replication and plaque formation. Even though changes only in the structural proteins in the virus mutant NS‐MBU‐1 likely maintain the small plaque phenotype, suggesting a crucial role of the NS proteins, additional mutations in the structural proteins while maintaining the mutations in the NS proteins may influence plaque size. These results suggest that attenuation characteristics are complex and are influenced by mutations in structural and NS proteins. Only certain mutation patterns confer attenuation properties.

### 3.4. NS‐MBU‐1 Is Significantly Less Virulent in Suckling Mice

A key feature of an attenuated virus is its reduced neurovirulence and lethality in newborn mice. To explore this further, we examined the effects of the mutant viruses compared to parental DENV3‐16562 and a saline solution (NSS) in newborn mice. The mice injected with NSS (*n* = 25) all survived, demonstrating baseline survival. In contrast, mice injected with the parental virus (*n* = 23) exhibited a survival rate of 73.91%, while those injected with the assembled DENV3‐MBU‐1 (*n* = 32) showed a significantly higher survival rate of 93.75% compared to the parental virus. The AST for mice injected with the parental virus was 19.04 ± 0.72 days, compared to 20.38 ± 0.44 days for those injected with DENV3‐MBU‐1. These findings demonstrated that DENV3‐MBU‐1 exhibited low neurovirulence, as expected (Figure 3A).

**FIGURE 3:**
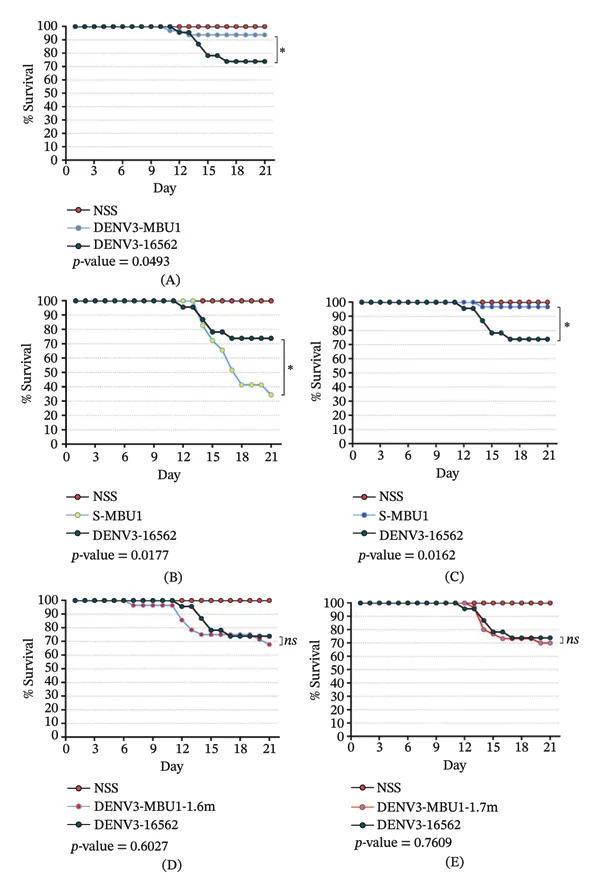
Survival curve of DENV3 mutants injected into newborn ICR mice. Two‐day‐old ICR mice were intracranially injected with 1000 PFU of the parental DENV3‐16562 (*n* = 23), the negative control NSS (*n* = 25), or one of the following: (A) DENV3‐MBU‐1 (*n* = 32) (*p*‐value = 0.0493), (B) S‐MBU‐1 (*n* = 29) (*p*‐value = 0.0177), (C) NS‐MBU‐1 (*n* = 30) (*p*‐value = 0.0162), (D) MBU‐1‐1.6 m (*n* = 28) (*p*‐value = 0.6027), or (E) MBU‐1‐1.7 m (*n* = 30) (*p*‐value = 0.7609). The mice were monitored daily for 21 days to assess morbidity and mortality. Survival rates were analyzed using the Log‐rank (Mantel–Cox) test. Asterisks indicate significant differences at *p* < 0.05, while “ns” denotes nonsignificant differences.

In addition, S‐MBU‐1, which is characterized by the reversion of NS mutations to their parental sequences while retaining mutations in structural regions, exhibited increased plaque size. In the study with newborn ICR mice (*n* = 29), the survival rate after 21 days was 34.48%, with an AST of 17.31 ± 0.60 days, which was significantly lower than that of the parental strain. This indicates a markedly increased neurovirulence for S‐MBU‐1 (Figure 3B). In contrast, mice infected with NS‐MBU‐1 (*n* = 30), which features reversions of structural mutations in addition to the retention of NS mutations, showed a survival rate of 96.67% by Day 21 with an AST of 20.73 ± 0.27 days (Figure 3C), similar to the results observed for the assembled DENV3‐MBU‐1. These findings showed that NS‐MBU‐1 and DENV3‐MBU‐1 mutant viruses are safe and support the pivotal role of NS proteins in low neurovirulence in mice.

To determine whether specific mutation patterns contribute to neurovirulence, MBU‐1‐1.6 m and MBU‐1‐1.7 m were examined. MBU‐1‐1.6 m, which contains a reversion of the NS3 protein mutation back to the parental nucleotide, and MBU‐1‐1.7 m, which contains a reversion of the NS4A mutation to the parental nucleotide, both exhibited increased neurovirulence, with mouse survival rates of 67.86% and 70.00%, respectively (Figure 3D, E). These rates are similar to the neurovirulence observed with the parental virus (73.91% survival) and are significantly more virulent than DENV3‐MBU‐1 (93.75% survival). The results indicate that both mutations are critical to the attenuation of this virus strain.

### 3.5. DENV3‐MBU‐1 and NS‐MBU‐1 Exhibit Lower Replication and Activate Higher IFN Response Than Parental DENV3‐16562 Virus in THP‐1 Cells

DENV3‐MBU‐1 and NS‐MBU‐1 were found to be less virulent in newborn mice, leading to an investigation of their attenuation properties. We hypothesized that these attenuated viruses might exhibit distinct replication kinetics patterns in immune cells compared to the parental DENV3‐16562. To evaluate this, viral growth kinetics were assessed in human monocyte THP‐1 cells. Both DENV3‐MBU‐1 and NS‐MBU‐1 exhibited lower replication than the parental virus, with the peak virus titer observed at Day 2, followed by a gradual decrease (Figure 4). To further investigate their ability to stimulate innate immune responses, the expression of IFN response genes, including IFNα and OAS, was analyzed in THP‐1 cells infected with the DENV3 mutants at the peak of infection. The results showed that DENV3‐MBU‐1 infection significantly increased the expression of both IFNα and OAS, whereas NS‐MBU‐1 infection increased OAS expression but not IFNα expression when compared to cells infected with the parental DENV3‐16562. Overall, these results suggest that DENV3‐MBU‐1 and NS‐MBU‐1 induce strong innate immune activation (Figure 5).

**FIGURE 4:**
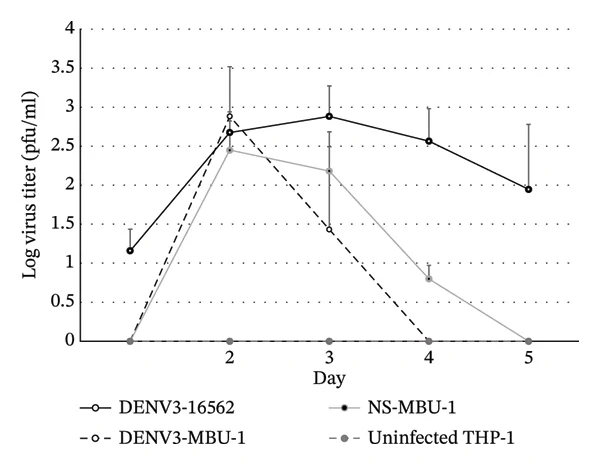
The kinetics of DENV3 mutant‐infected THP‐1 cells. THP‐1 cells were infected with DENV3 at MOI 2. Culture fluids were collected daily for five consecutive days postinfection. Viral titers were determined by plaque assay. The experiment was performed in triplicate. Error bars represent the standard deviation of the log‐transformed viral titer.

**FIGURE 5:**
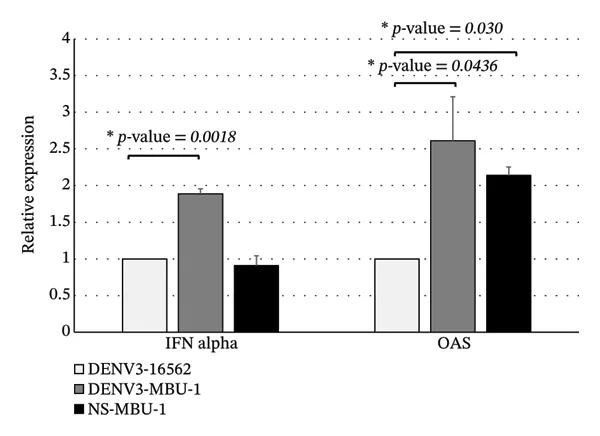
Relative expression of IFN‐related genes (IFNα and OAS) in DENV3 mutants infected THP‐1 cells. THP‐1 cells were infected at MOI 2 and harvested on Day 2 postinfection. Expression levels of IFN‐related genes were analyzed using real‐time RT‐PCR. The experiment was performed in triplicate. Error bars represent the standard deviation. Asterisks indicate significant differences by Student′s *t*‐test at *p* < 0.05.

Because some of the identified mutations are located in NS proteins (NS2A‐86, NS3‐90, and NS4A‐68), they may affect the formation, organization, or function of the viral replication complex. To explore this possibility, the intracellular distribution of double‐stranded RNA (dsRNA), a marker associated with viral RNA replication, was investigated by immunofluorescence assay in cells infected with DENV3‐MBU‐1, NS‐MBU‐1, and the parental DENV3 viruses. However, we could not clearly observe differences in dsRNA distribution among these viruses (Figure 6). Furthermore, to investigate whether the mutations affect viral RNA replication levels, RT‐qPCR assays targeting the 3′UTR of the viral genome were performed using THP‐1 cells infected the DENV3 mutants. Interestingly, only DENV3‐MBU‐1 showed significantly lower levels of RNA than both the parental DENV3 and NS‐MBU‐1. In contrast, no significant difference was observed between NS‐MBU‐1 and the parental DENV3 (Figure 7). This reduction of viral RNA may be supported by increased IFN‐α expression in mutant DENV3‐MBU‐1 (Figure 5), whereas mutant NS‐MBU‐1 showed neither a significant reduction in viral RNA levels nor an increase in IFN‐α expression.

**FIGURE 6:**
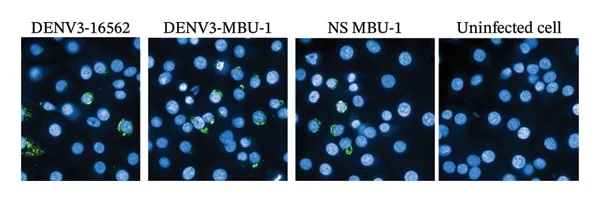
The immunofluorescence analysis of DENV3 parental, DENV3‐MBU‐1, and NS‐MBU‐1 viruses using an anti‐dsRNA antibody (green). Nuclei were stained with DAPI (blue).

**FIGURE 7:**
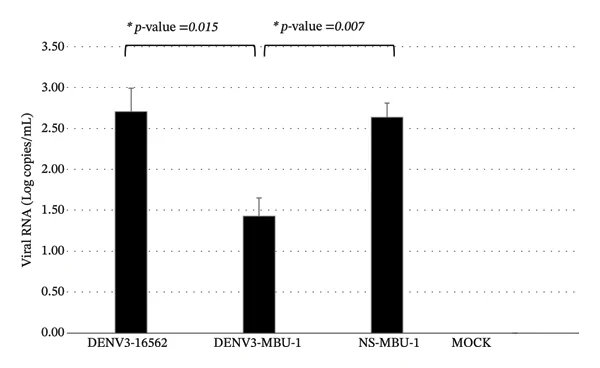
The RT‐qPCR targeting 3′UTR of THP‐1 cell‐infected DENV3 mutants. THP‐1 cells were infected with DENV3 at MOI 2 on Day 2 postinfection. Viral RNA was determined by RT‐qPCR. The experiment was performed in triplicate. Error bars represent the standard deviation of the log‐transformed viral RNA.

### 3.6. The Attenuated Phenotypes of DENV3‐MBU‐1 and NS‐MBU‐1 Appear Independent of Direct Disruption of the DC‐SIGN‐Binding Site

Because the identified mutations are located within EDIII of the envelope protein, a region involved in interactions with host cell receptors, molecular docking analysis between envelope and potential receptors may help determine whether these mutations affect receptor binding and contribute to the attenuated phenotype. Several cell‐surface molecules have been proposed to contribute to DENV attachment or entry into these cells, including the mannose receptor (MR) [26], glycosaminoglycans (GAGs) [27], DC‐SIGN (CD209) [28], β3 integrin, CD300a [29], TIM and TAM [30], and protein disulfide isomerase (PDI) [31].

Among these potential receptors, DC‐SIGN, MR, GAGs, and β3 integrin have previously been reported to bind to the DENV envelope. Studies have demonstrated that carbohydrate‐recognizing receptors, particularly MR and DC‐SIGN, can interact with glycans on the DENV envelope protein through their carbohydrate‐recognition domains. DC‐SIGN preferentially recognizes mannose residues within high‐mannose oligosaccharides and fucosylated glycans, whereas MR recognizes terminal mannose, fucose, and N‐acetylglucosamine residues. N‐linked glycosylation of the DENV envelope protein, particularly at N67, plays an important role in interactions with DC‐SIGN and MR [26, 32]. In addition to these carbohydrate‐dependent interactions, the DENV2 envelope protein has been reported to interact directly with β3 integrin, with putative binding regions located at residues C333–V347 and G381–K394 [33]. GAG binding has been associated with EDIII, with residues K291 and K295 identified as critical determinants of this interaction [34].

Of these host receptors, structural information is available for the interaction between the DENV3 envelope and DC‐SIGN. Therefore, we performed molecular docking and structural analyses to examine whether mutations located within or near EDIII, at E‐302, E‐327, and E‐392, might affect the interaction between the viral envelope protein and host cell receptors. Structural mapping showed that residues E‐302 and E‐327 are exposed on the surface of the envelope protein, whereas E‐392 is relatively less surface‐exposed (Figure 8A). However, none of these residues appeared to overlap with the predicted DC‐SIGN‐binding site (Figure 8B). These results suggest that the attenuated phenotype associated with these mutations is unlikely to result from direct disruption of the DC‐SIGN‐binding site.

**FIGURE 8:**
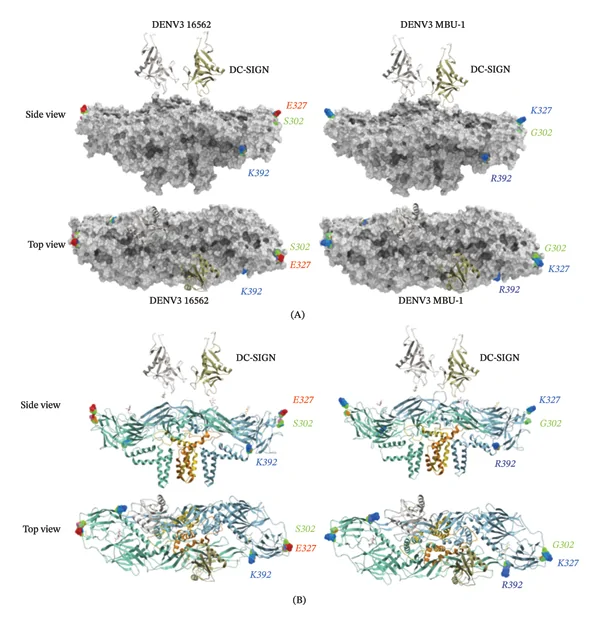
Crystal structure of the DENV3 envelope homodimer complex with the DC‐SIGN carbohydrate‐recognition domain CRD (PDB: 9MVE) was used to visualize the localization of amino acids that differ between DENV3‐16562 parental and DENV3‐MBU‐1 using ICM Pro 3.9 software. Top and side views are shown. The differing amino acid residues at Positions 302, 327, and 392 are highlighted in color. (A) Molecular surface views and (B) ribbon views of the DENV3 E protein.

## 4. Discussion

LAVs are produced by serial passages in non‐natural host cells, such as PGMK cells or PDK cells, until virulence is attenuated. Alternatively, LAVs can also be generated by recombinant techniques using reverse genetics if the attenuation markers are known. In this study, recombinant DENV3‐MBU‐1, generated by RT‐PCR‐amplified genomic segments assembled from DENV3‐16562 passaged into PGMK and PDK cells, showed attenuation properties both in vitro and in vivo. These characteristics of DENV3‐MBU‐1 include a small plaque phenotype and low neurovirulence in mice. Despite these observed attenuating properties, the key molecular markers responsible for attenuation remain to be characterized.

Comparative analysis of the genome sequences of DENV3‐MBU‐1 differed from the parental DENV3‐16562 by seven nonsynonymous substitutions in the prM (L95I), E (G302S, K327E, and R392K), NS2A (L86F), NS3 (K90Q), and NS4A (T68A) proteins. To investigate the significance of the individual point mutations, we first attempted to construct the full‐length infectious cDNA DENV3 clone. However, despite several efforts, we did not succeed in generating the full‐length DENV3 cDNA with accurate genome sequences. The plasmids obtained contained deletion mutations, so they could not produce infectious viruses. The main obstacle in these approaches is the instability of orthoflavivirus sequences in host systems, especially in *Escherichia coli* [35]. To overcome this problem, many viruses have been successfully generated using a bacteria‐free approach by transfecting the assembled fragments under the control of the CMV promoter. Examples include DENV [36], Zika virus [37], Enterovirus 71 [38], and porcine reproductive and respiratory syndrome virus [39]. In this study, we utilized the pDENV3‐ΔFL as a template to generate the mutant DENV3 by PCR‐amplified genomic assembled segments and then recovered in cocultured Vero:HEK‐293T.

In this study, we found that changes involving either single or multiple mutations affect the phenotypic characteristics and attenuation properties of the virus. The effects of mutations in the NS protein at positions NS2A‐86, NS3‐90, and NS4A‐68 either single mutations (MBU‐1‐1.6 m, MBU‐1‐1.7 m) or multiple mutations (S‐MBU‐1, MBU‐1‐345 m, MBU‐1‐346 m, MBU‐1‐347 m, MBU‐1‐3456 m, and D3‐MBU‐1‐3467 m) were observed to increase the plaque size of the viruses and influence their attenuation properties. This observation is supported by the results of the neurovirulence test in mice for S‐MBU‐1, MBU‐1‐1.6 m, and MBU‐1‐1.7 m, which showed an increase in virulence comparable to that of the parental DENV3. Interestingly, the S‐MBU‐1 strain, in which all three NS proteins were reverted to the parental virus, exhibited higher virulence than other mutants. Moreover, it was interesting to note that despite the Envelope Domain III (EDIII) mutations reverting to the parental genotype, the NS mutations were still able to maintain an attenuated phenotype in vivo, suggesting that they are critical. Collectively, these findings emphasize the critical role of the NS protein in DENV3‐MBU‐1 in maintaining its attenuating property.

The viral NS3 protein is a multifunctional protein with a proteolytic serine protease domain at the N‐terminus and an ATPase/helicase domain at the C‐terminus. NS3 plays an important role in viral genome replication and protein processing. The NS3 serine protease together with 40 amino acid residues of NS2B cleaves viral proteins that are critical for viral assembly [40–42]. The amino acid change K90Q of NS3 caused a radical replacement. From the crystal structure mapping data, this position is located adjacent to the orthoflavivirus conserved tryptophan residue (W89) on the linker peptide (aa 81–92) connecting the two subdomains (lobes) of the NS3‐serine protease domain. This protrusion of the linker peptide is located opposite the main catalytic groove and is not involved in the catalytic site of the enzyme [43, 44]. Although the protease activity of NS2B/NS3 has been extensively studied, many studies have focused on the NS2B–NS3 interaction and the catalytic triad of D75 and H51 on Subdomain I and S135 on Subdomain II [42, 45, 46]. However, site‐directed mutations at three positions next to K90Q on the linker portion substituting glutamic acid with alanine (E91A, E93A, and E94A) caused severely restricted viral replication and temperature sensitivity in DENV2 [47]. This suggests NS3‐90 may associate with viral replication.

During viral replication, the viral membrane proteins NS2A, NS4A, and NS4B form the viral replication complex [48]. NS4A is a small protein characterized by three α‐helix transmembrane segments with a cytosolic N‐terminus [49]. A mutation at position 6572 leads to an amino acid substitution from alanine to threonine at Position 68 of NS4A (A68T) in the parental strain. This position is located within the segment of the first transmembrane α‐helical domain (PTMD1) in the membrane of the endoplasmic reticulum (ER), which consists of hydrophobic amino acids that are crucial for the embedding of the protein in the lipid membrane of the ER [50]. The replacement of alanine (a nonpolar amino acid) by threonine (a polar amino acid) may lead to structural changes in the NS4A protein. Although no changes at this position have been previously reported, our study shows that the alteration of NS4A‐68 in MBU‐1‐1.7 m leads to an increase in plaque size and virulence. This finding emphasizes the importance of this position for virus attenuation.

DENV NS2A, a component of the viral replication complex, plays an important role in viral assembly and inhibition of host immune responses. Structurally, DENV NS2A comprises an N‐terminal segment (aa 1–68) located in the ER lumen, five transmembrane segments (aa 69–209) embedded in the ER membrane, and a C‐terminal region (aa 210–218) located in the cytosol. The NS2A‐86 position is predicted to be part of the transmembrane α‐helix domain 3 (PTMD3). Although it was not possible to recover the virus with the MBU‐1‐1.5 m (NS2A‐L86F) mutation, replacement of residues within this PTMD3 region of DENV2 with glutamic acid (R84E) or alanine (R84A) has been shown to inhibit viral RNA synthesis and viral assembly [51]. Further studies are needed to clarify the effects of this mutation.

To explore whether the attenuated phenotypes observed in some DENV3 mutants might be associated with altered interactions with host cell receptors, molecular docking analysis was performed using the available structural information for the DENV3 E protein and the DC‐SIGN cellular receptor. The findings suggest that these mutations are unlikely to cause attenuation through direct disruption of the DC‐SIGN‐binding site. However, indirect effects on envelope protein conformation or dynamics cannot be excluded.

The potential effects of these mutations on interactions with β3 integrin and GAGs could not be evaluated in this study because an appropriate structural model was not available for molecular docking analysis. Further studies using structural modeling and receptor‐binding assays may be required to determine whether these mutations alter receptor usage or binding efficiency.

Molecular determination of attenuation has been reported in the DENV3 vaccine candidate KD‐382, an attenuated virus derived from PGMK30/PDK4. While seven mutations were identified in DENV3‐MBU‐1 in this study, the DENV3 vaccine candidate KD‐382 had 13 mutations spanning several parts of the viral genome (pr, M, E, NS2A, NS2B, NS3, NS4, and NS5). Interestingly, four mutations were found at similar positions between the DENV3 vaccine seed in KD‐382 and DENV3‐MBU‐1. Two in EDII at aa 302, 392, and two in NS proteins (NS2A aa86 and NS3 aa90) [17]. In this study, single mutations of E‐G302S and NS3‐K90Q were investigated. This result emphasizes that these mutations can attenuate DENV3‐16562.

Furthermore, we hypothesize that mutations in conserved nucleotides affect both viral fitness and virulence. In this study, sequence conservation and variation were demonstrated by aligning 79 complete genome sequences of DENV3 from the GenBank database. The results showed that Positions 1838 (E‐302), 1913 (E‐327), 3725 (NS2A‐86), 4781 (NS3‐90), and 6572 (NS4A‐68) showed high conservation, while the other nucleotides showed some variation (Supporting Tables S3 and S4). Altogether, the results suggest that these mutations play an important role in virus attenuation.

While wild‐type DENV typically evades innate immunity by inhibiting Type I IFN signaling [52], these mutants appear to induce an innate immune response by activating the expression of IFNα and OAS. These findings suggest that the enhanced IFN‐α response induced by DENV3‐MBU‐1 may contribute to the reduced efficiency of viral genome replication. In contrast, no significant change in IFN‐α expression in NS‐MBU‐1 may partly explain why its viral RNA level remained comparable to that of the parental DENV3.

The ability of an attenuated vaccine strain to stimulate an innate immune response was demonstrated by Pintado Silva J and colleagues [53]. Their study showed that the DENV4 vaccine strain, a component of NIAID’s tetravalent TV003 vaccine, exhibited lower replication and infection efficiency in monocyte‐derived dendritic cells compared to the wild‐type virus. Moreover, this DENV4 vaccine strain significantly induced IFNα2 and the proinflammatory cytokine IP‐10. These findings support our study, emphasizing the ability of attenuated vaccine strains to trigger innate immune responses and contribute to their immunogenic properties. Further studies are needed to investigate the extent to which DENV3‐MBU‐1 and NS‐MBU‐1 stimulate innate immune and proinflammatory responses, and to evaluate their immunogenicity in vivo.

Undoubtedly, the development of vaccines against DENV is very complex due to its serotypes and sequence variability. In this study, we determine the molecular aspects related to viral attenuation. Many mutants were generated. Although we could not generate all mutants, the combination mutations still provided insights into the importance of NS mutations and EDIII mutations. Here, we found that a specific pattern rather than a single mutation affects overall viral attenuation. This finding provides important information for the development of a DENV vaccine.

## 5. Conclusions

Our study bridged the gap in identifying the key molecular markers of DENV3‐MBU‐1 to inform future LAV dengue vaccine development. We highlighted the importance of EDIII and NS protein mutations as the attenuating mutations of this vaccine candidate strain. Future vaccine development should consider the interplay of structural and NS mutations in viral attenuation.

## Author Contributions

Conceptualization: Prasert Auewarakul, Promsin Masrinoul, Sutee Yoksan; methodology: Panumas Sun‐Arlee, Prasert Auewarakul, Anan Jongkaewwattana, and Promsin Masrinoul; investigation: Panumas Sun‐Arlee, Ekkarat Rodpai, Kunjimas Ketsuwan, Duangnapa Wanlayaporn, Sasiporn Ruangdachsuwan, Jukrapun Komaikul, Thitigun Jaimipuk, Sanjira Juntarapornchai, Surat Punyahathaikul, Chompunuch Boonarkart, and Promsin Masrinoul; funding acquisition: Promsin Masrinoul; resources: Sutee Yoksan, Prasert Auewarakul, and Anan Jongkaewwattana; writing–original draft: Panumas Sun‐Arlee, Promsin Masrinoul; writing–review and editing: Panumas Sun‐Arlee, Promsin Masrinoul, Sutee Yoksan, Anan Jongkaewwattana, and Prasert Auewarakul.

## Funding

This research was supported by the National Science and Technology Development Agency (NSTDA) (Grant No. FDA‐CO‐2560‐3819‐TH).

## Disclosure

All authors read and approved the final manuscript.

## Conflicts of Interest

The authors declare no conflicts of interest.

## Supporting Information

Additional supporting information can be found online in the Supporting Information section.

## Supporting information


**Supporting Information** Supporting Figure S1. Chromatograms of single‐point virus mutants. Supporting Figure S2. Chromatograms of multiple point virus mutants. Supporting Table S1. Primer sets used for PCR amplification of DENV‐3 subgenomic fragments. Supporting Table S2. Primer set used for mutant construction. Supporting Table S3: Complete DENV‐3 genome sequences used in sequence conservation and variation analyses. Supporting Table S4: Summary of nucleotide conservation and variation identified through the analysis of complete DENV‐3 genome sequences.

## Data Availability

The datasets generated and/or analyzed during the current study are available in the National Center for Biotechnology Information (NCBI) GenBank repository under the following accession numbers: DENV3‐MBU‐1 (PQ895348), NS‐MBU1 (PQ895349), S‐MBU1 (PQ895350), MBU‐1‐1.1 m (PQ895351), MBU‐1‐1.2 m (PQ895352), MBU‐1‐1.4 m (PQ895353), MBU‐1‐1.6 m (PQ895354), MBU‐1‐1.7 m (PQ895355), MBU‐1‐34 m (PQ895356), MBU‐1‐345 m (PQ895357), MBU‐1‐346 m (PQ895358), MBU‐1‐347 m (PQ895359), MBU‐1‐3456 m (PQ895360), and MBU‐1‐3467 m (PQ895361).
