# Supplementary material for: Impact of Specific Pattern Mutations on Plaque Formation and Attenuation Properties of Dengue Virus Serotype 3
Source: Adv Virol. 2026 Sep 29;2026:9239020. doi: 10.1155/av/9239020 (PMC13624709; doi:10.1155/av/9239020)
Supplement: Supplementary file 1 [file AV-2026-9239020-s001.docx]

**Impact of Specific Pattern Mutations on Plaque Formation and Attenuation Properties of Dengue Virus Serotype 3**

**Supplementary**

**MBU-1-1.1m**

**MBU-1-1.2m**

**MBU-1-1.4m**

**MBU-1-1.6m**

**MBU-1-1.7m**

**Supplementary Figure S1.** Chromatograms of single point mutants with successful assembly. (Top to bottom) Chromatograms of successful mutation or retention of nucleotides of MBU-1-1.1m, MBU-1-1.2m, MBU-1-1.4m, MBU-1-1.6m, MBU-1-1.7m. MBU-1-1.3m and MBU-1-1.5m were not successfully assembled. Red arrows indicate specific nucleotides of interest (the seven non-synonymous substitutions between DENV3-16562 and DENV3-MBU-1).

**MBU-1-34m**

**MBU-1-345m**

**MBU-1-346m**

**MBU-1-347m**

**MBU-1-3456m**

**Supplementary Figure S2.** Chromatograms of multiple point mutants with successful assembly. (Top to bottom) Chromatograms of successful mutation or retention of nucleotides of MBU-1-34m, MBU-1-345m, MBU-1-346m, MBU-1-347m, MBU-1-3456m. Red arrows indicate specific nucleotides of interest (the seven non-synonymous substitutions between DENV3-16562 and DENV3-MBU-1).

**Supplementary Table S1.** Primer sets used for PCR amplification of DENV-3 subgenomic fragments.

| Primer | Sequence (5' → 3') | Position | Product size (bp) |
| --- | --- | --- | --- |
| DENV3_Ffr1 | CTATAGGGAGACCCAAGCTGGCTAGCAGTTGTTAGTCTACGTGGA CCGACAAGAAC | 1 | 2121 |
| DENV3_Rfr1_C_MBU1 | ATCGAGCTTCCCTTC***C***TGTACCAGTTGATTTT | 2,121 |  |
| DENV3_Ffr2_G_MBU1 | AAAATCAACTGGTACA***G***GAAGGGAAGCTCGAT | 2,090 | 2707 |
| DENV3_Rfr2_T_MBU1 | CCTCCTCCCCCTTTT***T***CCATTGTGCACTCAA | 4,796 |  |
| DENV3_Ffr3_A_MBU1 | TTGAGTGCACAATGG***A***AAAAGGGGGAGGAGG | 4,766 | 1822 |
| DENV3_Rfr3_T_MBU1 | TCAAGAAGAGGCTTG***T***TCCACCTGTTAACAA | 6,587 |  |
| DENV3_Ffr4_A_MBU1 | TTGTTAACAGGTGGA***A***CAATGCTCTTCTTGA | 6,557 | 1966 |
| DENV3_Rfr4 | TCATGGAGGAGGCTG***A***GCCTGTGGC | 8,522 |  |
| DENV3_Ffr5 | GCCACAGGCTCAGCCTCCTCCATGA | 8,498 | 2199 |
| DENV3_Rfr5 | GAATGTTGCCCAGCCGGCGCCAGCGAGGAGGCTGGGACCATGCCGGCAGAACCTGTTGATTCAAC | 10,696 |  |

**Supplementary Table S2.** Primer set used for mutant construction

| Primer | Sequence (5' → 3') | Position |
| --- | --- | --- |
| CMV_DENV3_fr1_F | GCTCAA TAT TGG CCA TTA GCC | CMV |
| *A*TA719_F | AGTGGCG***A*TA**GCTCCCCATGTTGGCATGG | 719 |
| *A*TA*71*9_R | GGAGC**TA*T***CGCCACTGATCTCTTATCGCG | 719 |
| *T*TA719_F | AGTGGCG***T*TA**GCTCCCCATGTTGGCATGG | 719 |
| *T*TA*71*9_R | GGAGC**TA*A***CGCCACTGATCTCTTATCGCG | 719 |
| *A*GT1838_F | GTGCTTG***A*GT**AGCTTTGTGTTGAAGAAAGAAGTCT | 1838 |
| *A*GT1838_R | AAGCT**AC*T***CAAGCACATTGCATAGCTCATCCC | 1838 |
| *G*GT1838_F | GTGCTTG***G*GT**AGCTTTGTGTTGAAGAAAGAAGTCT | 1838 |
| *G*GT1838_R | AAGCT**AC*C***CAAGCACATTGCATAGCTCATCCC | 1838 |
| *A*AA1913_F | CAAAGGG***A*AA**GATGCACCCTGCAAGATTCCT | 1913 |
| *A*AA1913_R | GCATCTT*T*CCCTTTGTACTCAACCTTAATGAGT | 1913 |
| *G*AA1913_F | CAAAGGG***G*AA**GATGCACCCTGCAAGATTCCT | 1913 |
| *G*AA1913_R | GCATC**TT*C***CCCTTTGTACTCAACCTTAATGAGT | 1913 |
| A*A*G2106_F | AAAATCAACTGGTACA***A***GAAGGGAAGCTCGAT | 2106 |
| A*A*G2106_R | ATCGAGCTTCCCTTC***T***TGTACCAGTTGATTTT | 2106 |
| A*G*G2106_F | AAAATCAACTGGTACA***G***GAAGGGAAGCTCGAT | 2106 |
| A*G*G2106_R | ATCGAGCTTCCCTTC***C***TGTACCAGTTGATTTT | 2106 |
| *T*TC3725_F | TCAGCCA***T*TC**CTGGCTTTGGGATTCTTCCTG | 3725 |
| *T*TC3725_R | GCCAG**GA*A***TGGCTGAATTTTAAATGTTGCAA | 3725 |
| *C*TC3725_F | TCAGCCA***C*TC**CTGGCTTTGGGATTCTTCCTG | 3725 |
| *C*TC3725_R | GCCAGGA*G*TGGCTGAATTTTAAATGTTGCAA | 3725 |
| *A*AA4781_F | ACAATGG***A*AA**AAGGGGGAGGAGGTGCAG | 4781 |
| *A*AA4781_R | CCCTT**TT*T***CCATTGTGCACTCAATCTCCATCC | 4781 |
| *C*AA4781_F | ACAATGG***C*AA**AAGGGGGAGGAGGTGCAG | 4781 |
| *C*AA4781_R | CCCTT**TT*G***CCATTGTGCACTCAATCTCCATCC | 4781 |
| *A*CA6572_F | AGGTGGA***A*CA**ATGCTCTTCTTGATATCAGGTAAAG | 6572 |
| *A*CA6572_R | AGCAT**TG*T***TCCACCTGTTAACAAGATCATCAG | 6572 |
| *G*CA6572_F | AGGTGGA***G*CA**ATGCTCTTCTTGATATCAGGTAAAG | 6572 |
| *G*CA6572_R | AGCAT**TG*C***TCCACCTGTTAACAAGATCATCAG | 6572 |
| DENV_Fix3_F | GGAAGTGGAATTTTCGTCACTAATGAGGTCC | pSM |
| DENV_Fix3_R | GTGACGAAAATTCCACTTCCACATTTGAGTTC | pSM |
| BGH_DENV3_fr5_R | CCATAGAGCCCACCGCATCC | BGH |

**Supplementary Table S3:** Complete DENV-3 genome sequences from the GenBank database used in sequence conservation and variation analyses.

| No. | DENV3 | country | year |
| --- | --- | --- | --- |
| 1 | DENV3 16562 | Philippines | 1964 |
| 2 | DENV3 H87 | Philippines | 1987 |
| 3 | DENV3 D17011 | Philippines | 2017 |
| 4 | DENV3 ThD3_0007_87 | Thailand | 1987 |
| 5 | DENV3 ThD3_0010_87 | Thailand | 1993 |
| 6 | DENV3 ThD3_0055_93 | Thailand | 1993 |
| 7 | DENV3 ThD3_0104_93 | Thailand | 1998 |
| 8 | DENV3 ThD3_1687_98 | Thailand | 1998 |
| 9 | DENV3_C0360/94 | Thailand | 1994 |
| 10 | DENV3_D83-144 | Thailand | 1983 |
| 11 | DENV3 23GZ10604_D1_THA_F | Thailand | 2023 |
| 12 | DENV3 8561 | Thailand | 2010 |
| 13 | DENV3 CNR_15418 | Thailand | 2012 |
| 14 | DENV3/IPC/BID-V3820/2006 | Cambodia | 2006 |
| 15 | DENV3 MS12007786 | Malaysia | 2014 |
| 16 | DENV3_D3/SG/05K4648DK1/2005 | Singapore | 2005 |
| 17 | DENV3_D3/SG/SS710/2004 | Singapore | 2004 |
| 18 | DENV3_D3/SG/05K4144DK1/2005 | Singapore | 2005 |
| 19 | DENV3_D3/SG/05K4168DK1/2005 | Singapore | 2005 |
| 20 | DENV3 SG(EHI)D3/05416Y13 | Singapore | 2018 |
| 21 | DENV3 SG(EHI)D3/09568Y18 | Singapore | 2013 |
| 22 | DENV3_58760/2005 | Indonesia | 2005 |
| 23 | DENV3_Sleman/78 | Indonesia | 1978 |
| 24 | DENV3 Indonesia/201610225 | Indonesia | 2016 |
| 25 | DENV3 MKS-WS78 | Indonesia | 2010 |
| 26 | DENV-3/VN/BID-V1008/2006 | Vietnam | 2006 |
| 27 | DENV-3/VN/BID-V1009/2006 | Vietnam | 2006 |
| 28 | DENV-3/VN/BID-V1017/2007 | Vietnam | 2007 |
| 29 | DENV3/VN/BID-V1014/2006 | Vietnam | 2006 |
| 30 | DENV3_ZJYW2009 | China | 2009 |
| 31 | DENV3_80-2 | China | 1980 |
| 32 | DENV3_13GDZDVS30E | China | 2013 |
| 33 | DENV3 13GDZDVS30B | China | 2013 |
| 34 | DENV3 GZ1D3 | China | 2009 |
| 35 | DENV3 HN/2013/107 | China | 2013 |
| 36 | DENV3 D17139 | China | 2017 |
| 37 | DENV3_HN/2013/110 | Hongkong | 2013 |
| 38 | DENV3_99TW628 | Taiwan | 1999 |
| 39 | DENV3 98TW407 | Taiwan | 1998 |
| 40 | DENV3_98TW414 | Taiwan | 1998 |
| 41 | DENV3 Bangladesh-UF-1/2019 | Bangladesh | 2019 |
| 42 | DENV3_D3/H/IMTSSA-SRI/2000/1266 | SriLanka | 2000 |
| 43 | DENV3 SLS0023 | SriLanka | 2024 |
| 44 | DENV3_CDBT_TU_NP224 | Nepal | 2022 |
| 45 | DENV3_DEL-72_India | India | 2008 |
| 46 | DENV3 Rajasthan.India/DMRC/Balotra87/2013 | India | 2013 |
| 47 | DENV3 THSTI-TRC-DENV3-10 | India | 2018 |
| 48 | DENV3_D3/Pakistan/45251/2009 | Pakistan | 2009 |
| 49 | DENV3_D3/Pakistan/43298/2006 | Pakistan | 2006 |
| 50 | DENV3_Cairns2008 | Australia | 2008 |
| 51 | DENV3_D3BR/RP1/2003 | Brazil | 2003 |
| 52 | DENV3_BR DEN3 98-04 | Brazil | 2004 |
| 53 | DENV3_BR DEN3 290-02 | Brazil | 2002 |
| 54 | DENV3/CO/BID-V3405/2007 | Columbia | 2007 |
| 55 | DENV3_D3/H/IMTSSA-MART/1999/1243 | Martinique | 1999 |
| 56 | DENV3_PF90/3050 | French Polynesia | 1990 |
| 57 | DENV3_PF90/3056 | French Polynesia | 1990 |
| 58 | DENV3_PF92/4190 | French Polynesia | 1992 |
| 59 | DENV3_Cuba_20_2002 | Cuba | 2002 |
| 60 | DENV3_Cuba_73_2001 | Cuba | 2001 |
| 61 | DENV3_Cuba_492_2001 | Cuba | 2001 |
| 62 | DENV3_Cuba_21_2002 | Cuba | 2002 |
| 63 | DENV-3/NI/BID-V7663/2011 | Nicaragua | 2011 |
| 64 | DENV-3/NI/BID-V7658/2012 | Nicaragua | 2012 |
| 65 | DENV-3/PE/BID-V6262/2007 | Nicaragua | 2007 |
| 66 | DENV-3/VE/BID-V2479/2007 | Venezuela | 2007 |
| 67 | DENV-3/VE/BID-V2456/2001 | Venezuela | 2001 |
| 68 | DENV3_D3/Hu/TL129NIID/2005 | EastTimor | 2005 |
| 69 | DENV3_D3/Hu/TL029NIID/2005 | EastTimor | 2005 |
| 70 | DENV3_NC96/181196-4690 | New Caledonia | 1996 |
| 71 | DENV3_NC95/240295-418 | New Caledonia | 1995 |
| 72 | DENV3 07178/DENV3/FG/2021-01-07 | French Guiana | 2021 |
| 73 | DENV3 01004/DENV3/FG/2023-09-01 | French Guiana | 2023 |
| 74 | DENV3 Jeddah-2014 | Saudi Arabia | 2014 |
| 75 | DENV3 SYMAV-07/Gabon/2016 | Gabon | 2016 |
| 76 | DENV-3/PE/BID-V7070/2008 | Peru | 2008 |
| 77 | DENV-3/PE/BID-V7082/2005 | Peru | 2005 |
| 78 | DENV3 D3PY/SUS/2003 | Paraguay | 2003 |
| 79 | DENV3 449334 | Colombia | 2015 |

**Supplementary Table S4:** Summary of nucleotide conservation and variation identified through the analysis of 79 complete DENV-3 genome sequences retrieved from the GenBank database **(Supple. S3)**

| **Genome position** | **Protein position** | **Nucleotide (conservation rate)** |
| --- | --- | --- |
| 719 | prM-95 | T(97.5%), C (2.5%) |
| 1838 | E-302 | A (100%) |
| 1913 | E-327 | G (100%) |
| 2106 | E-392 | A(97.5%), G (2.5%) |
| 3725 | NS2A-86 | T (100%) |
| 4781 | NS3-90 | C (100%) |
| 6572 | NS4A-68 | G (100%) |
